# Predicting cochlear dead regions in patients with hearing loss through a machine learning-based approach: A preliminary study

**DOI:** 10.1371/journal.pone.0217790

**Published:** 2019-06-03

**Authors:** Young-Soo Chang, Heesung Park, Sung Hwa Hong, Won-Ho Chung, Yang-Sun Cho, Il Joon Moon

**Affiliations:** 1 Department of Otorhinolaryngology–Head and Neck Surgery, Korea University College of Medicine, Korea University Ansan Hospital, Ansan, Republic of Korea; 2 Department of Digital Health, SAIHST, Sungkyunkwan University, Seoul, Republic of Korea; 3 Hearing Research Laboratory, Samsung Medical Center, Seoul, Republic of Korea; 4 Department of Otorhinolaryngology–Head and Neck Surgery, Samsung Changwon Hospital, Sungkyunkwan University School of Medicine, Changwon, Republic of Korea; 5 Department of Otorhinolaryngology–Head and Neck Surgery, Samsung Medical Center, Sungkyunkwan University School of Medicine, Seoul, Republic of Korea; Medical University Hannover; Cluster of Excellence Hearing4all, GERMANY

## Abstract

We propose a machine learning (ML)-based model for predicting cochlear dead regions (DRs) in patients with hearing loss of various etiologies. Five hundred and fifty-five ears from 380 patients (3,770 test samples) diagnosed with sensorineural hearing loss (SNHL) were analyzed. A threshold-equalizing noise (TEN) test was applied to detect the presence of DRs. Data were collected on sex, age, side of the affected ear, hearing loss etiology, word recognition scores (WRS), and pure-tone thresholds at each frequency. According to the cause of hearing loss as diagnosed by the physician, we categorized the patients into six groups: 1) SNHL with unknown etiology; 2) sudden sensorineural hearing loss (SSNHL); 3) vestibular schwannoma (VS); 4) Meniere's disease (MD); 5) noise-induced hearing loss (NIHL); or 6) presbycusis or age-related hearing loss (ARHL). To develop a predictive model, we performed recursive partitioning and regression for classification, logistic regression, and random forest. The overall prevalence of one or more DRs in test ears was 20.36% (113 ears). Among the 3,770 test samples, the overall frequency-specific prevalence of DR was 6.7%. WRS, pure-tone thresholds at each frequency, disease type (VS or MD), and frequency information were useful for predicting DRs. Sex and age were not associated with detecting DRs. Based on these results, we suggest possible predictive factors for determining the presence of DRs. To improve the predictive power of the model, a more flexible model or more clinical features, such as the duration of hearing loss or risk factors for developing DRs, may be needed.

## Introduction

A cochlear dead region (DR) is defined as a region in the cochlea where the inner hair cells (IHCs) and/or neurons lose normal function at a related frequency. Detecting the presence of DRs is important in clinical practice. A previous study had reported that DRs are associated with potentially poor hearing thresholds on follow-up audiograms in patients with sudden sensorineural hearing loss (SSNHL) [1]. Since it is debatable whether the presence of DRs, especially in high frequencies, is associated with hearing aid fitting and amplification selection [2–6], studies to detect the presence of DRs and to reveal their role continue.

The threshold-equalizing noise test proposed by Moore et al. [7] is designed to detect the presence of a cochlear dead region (DR) in a clinical setting. The test consists of measuring the threshold for detecting a pure tone presented in a background noise, called the threshold-equalizing noise (TEN). When the pure-tone signal frequency falls in a DR, the signal will only be detected when it produces sufficient basilar membrane vibration in a remote region of the cochlea where there are surviving IHCs and neurons. The amount of vibration produced by the tone in this remote region will be less than that of the dead region, and so the noise will be very effective in masking the signal. In patients with DRs, the TEN-masked threshold at a specific frequency associated with the DR is expected to be higher than in individuals with normal hearing [8]. When the TEN-masked threshold is at least 10 dB higher than the TEN level and 10 dB higher than the listener’s unmasked threshold, the condition is indicative of a cochlear DR [7, 9]. The TEN test can be categorized into two versions. An earlier version was calibrated according to a dB sound pressure level (SPL) and is referred to as the TEN (SPL) test [10]. A later version was designed to provide approximately the same masked pure-tone thresholds in dB HL for wide frequencies (500–4000 Hz) and is referred to as the TEN (HL) test [7].

Several studies have reported reliable indicators of DRs based on detection by TEN tests [2, 11, 12]. Hearing thresholds of specific frequencies [2, 9, 11] and hearing impairments with slopes of at least 20 dB/octave [12] have been reported as possible indicators of DRs. However, it is still challenging to predict DRs in patients with hearing loss based on clinical and audiologic findings [2].

Machine learning (ML) is evolving with advances in computing power. Numerous ML methodologies have been developed and adopted in clinical practice, and several studies have demonstrated the successful application of ML models as effective predictive models in clinical practice [13–15]. However, no studies have successfully applied ML in the audiologic field to develop predictive models. Herein, we propose a machine learning (ML)-based model for predicting DRs in patients with hearing loss due to various diseases.

## Materials and methods

### Subjects

Patients diagnosed with SNHL who visited an outpatient clinic and agreed to participate were enrolled. A total of 555 ears from 380 patients (3,770 test samples) were included in the study.

Based on clinical findings from a review of medical charts by one experienced otolaryngologist, patients were categorized into one of the following six disease groups: 1) SNHL with unknown etiology, defined as hearing loss with less than 10 dB of air-bone gap, and could not be classified into another group; 2) SSNHL, defined as hearing loss greater than 30 dB in three continuous frequencies within three days after initial diagnosis, and enrolled in the study within six months after diagnosis; 3) vestibular schwannoma, defined as hearing loss with identifiable ipsilateral vestibular schwannoma on magnetic resonance imaging (VS); 4) Meniere’s disease, defined as hearing loss that meets the diagnostic criteria of Meniere`s disease (MD) [16]; 5) noise-induced hearing loss, defined as hearing loss due to excessive noise exposure, either acute exposure or chronic occupational exposure (NIHL); and 6) presbycusis or age-related hearing loss, defined as bilateral symmetric sensorineural deafness in patients age 50 years or older with a typical downslope audiometric pattern above 1000 Hz (ARHL) [17].

The medical information of patients diagnosed with sensorineural hearing loss (SNHL) was gathered over a period from September 2010 to May 2015. In the present study, we performed the TEN (HL) test on all patients. The detailed methodology of the TEN (HL) test is described below. Medical records, audiology results, and TEN (HL) tests were retrospectively reviewed. Patients with any signs of acute infection were excluded. All participants provided their written informed consent to participate in this study. The Institutional Review Board of Samsung Medical Center approved this study (IRB No. 2010-03-004). This study was carried out in accordance with approved guidelines.

Data were collected on sex, age, side of the affected ear, diagnostic disease of the hearing loss, word recognition scores (WRS), and pure-tone thresholds at each frequency. WRS were obtained using 50 monosyllabic words 30 dB above the speech reception threshold of the test ear [18]. The monosyllabic words were selected based on word familiarity, phonetical dissimilarity, normal sampling of Korean speech sounds, and homogeneity with respect to intelligibility, and the percent correct scores were obtained [19].

### TEN (HL) test

The TEN (HL) test was performed following the protocol described by Moore et al. [7]. For conducting the TEN (HL) test, a pure-tone and a threshold-equalizing noise were played through a CD player connected to an audiometer that was calibrated to an audio player (RCD-M75U; Samsung, Suwon, Korea). The threshold-equalizing noise has been shaped so that the masked threshold of a pure tone is the same for all frequencies from 250 to 10,000 Hz in normal-hearing subjects. The TEN (HL) test was conducted by an experienced audiologist in a double-walled sound proof booth. An ER-3A insert phone was used as the transducer. A TEN level of 10 dB and above the hearing threshold at a given frequency region was selected to obtain a reliable masking effect. The TEN presentation level never exceeded more than 95 dB HL [7]. Subjects were asked to detect the introduced pure-tone in the TEN, and an examiner helped the patient find the masked threshold in a 2-dB step size by using the modified Hughson–Westlake procedure [20]. The pure-tone and TEN thresholds were obtained at 0.5, 0.75, 1, 1.5, 2, 3, and 4 kHz. At each specific frequency, when the threshold of the test tone in the TEN was 10 dB or more above the TEN level and the threshold of the test tone in the TEN was 10 dB or more above the absolute threshold, a DR was diagnosed at that frequency [7]. In patients where the TEN (HL) level could not be increased enough to elevate the absolute threshold by 10 dB or more, the results were considered inconclusive [9]. These patients were also included in the analysis and assessed as the non-DR frequency in the present study [20].

### Model development

We developed a predictive model in two steps. In the first step, we performed recursive partitioning and regression to build a classification tree (CT). Recursive partitioning creates a decision tree that attempts to correctly classify members of a population by splitting the populations into sub-populations based on several dichotomous, independent variables. The process is termed recursive because each sub-population may be split an indefinite number of times until a particular stopping criterion is reached (minimal split number = 20). In this process, the model suggests a break point that splits the population into sub-populations and is used for predicting the results. We applied this break point to the continuous variable to bag the data and then preprocessed the data in preparation for the second step.

In the second step, we used logistic regression (LR) and random forest (RF) to construct binary classification models. Using these two models, we evaluated the clinical significance in terms of screening. To achieve better screening for the presence of DRs, we evaluated the performance of LR models with a probability of 0.1. The theoretical bases of the LR and RF algorithms have been described in previous studies [21]. LR measures the relationship between categorical dependent variables and one or more independent variables by using probability scores based on the logit function [14]. The probability in LR indicates the result of the logistic function, which gives outputs between 0 and 1 for all values. The break point of the probability can be adjusted according to the distribution of data. We set a probability of 0.1 as the predictive probability of DR. RF is assembled by constructing a large number of decision trees with random subsets of model parameters that are used to define each split in the tree, and comprises many classification trees, the bagging idea, and a random selection of features [22]. In addition, the internal variance estimation method is used to measure variable importance, which indicates the variables of highest importance for splitting data. The internal variance estimation method generates predictions and estimates the variance parameters via one procedure by taking the mean and variance of the predictions generated by the trees, thus offering more computational efficiency than the external variance estimation method.

The models were constructed and tested using R (version 3.4.4, R Foundation for Statistical Computing, http://www.r-project.org/) with the rpart and caret packages [23, 24].

### Statistical analysis and model evaluation methods

Descriptive analysis was used to evaluate the prevalence of DR. To determine the distribution differences of DR according to disease type, the SNHL with unknown etiology group was used as the reference distribution of DR in each frequency, and Pearson’s Chi-squared test was performed. All analyses were performed using the R software (version 3.4.4, R Foundation for Statistical Computing, http://www.r-project.org/). A two-sided p-value <0.05 was considered statistically significant.

The accuracy of each model was quantified by calculating the sensitivity, specificity, positive predictive value (PPV), and negative predictive value (NPV). In addition, we used a 10-fold cross-validation approach to train (9 folds) and test (one fold) the LR and CT models. Model performance was evaluated through a 10-fold cross validation to ensure minimized bias, and the accuracy of each model was calculated.

## Results

The overall prevalence of one or more DRs in the test ears evaluated using the TEN (HL) test was 20.36% (113 ears). Among the 3,770 test samples, the overall frequency-specific prevalence of DR was 6.7%. The mean age (± standard deviation) of the study population was 56.4 ± 13.8 years. Two hundred and five patients were tested in one ear, and 175 patients were tested in both ears. Descriptive statistics of the study population are listed in Table 1.

**Fig 1:**
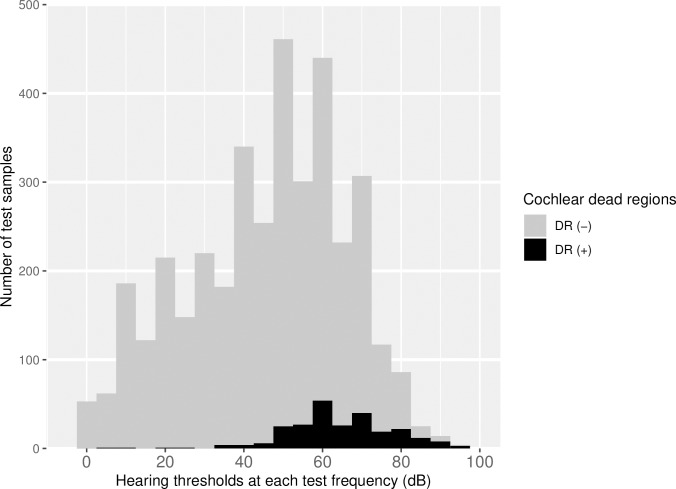
The distribution of cochlear dead regions according to the hearing thresholds at each frequency. The grey bar denotes the number of samples tested and the black bar denotes the number of cochlear dead regions. Note that the y-axis indicates the number of test samples. DR (+), diagnosed as a cochlear dead region by TEN (HL) test; DR (-), not a cochlear dead region.

**Fig 2:**
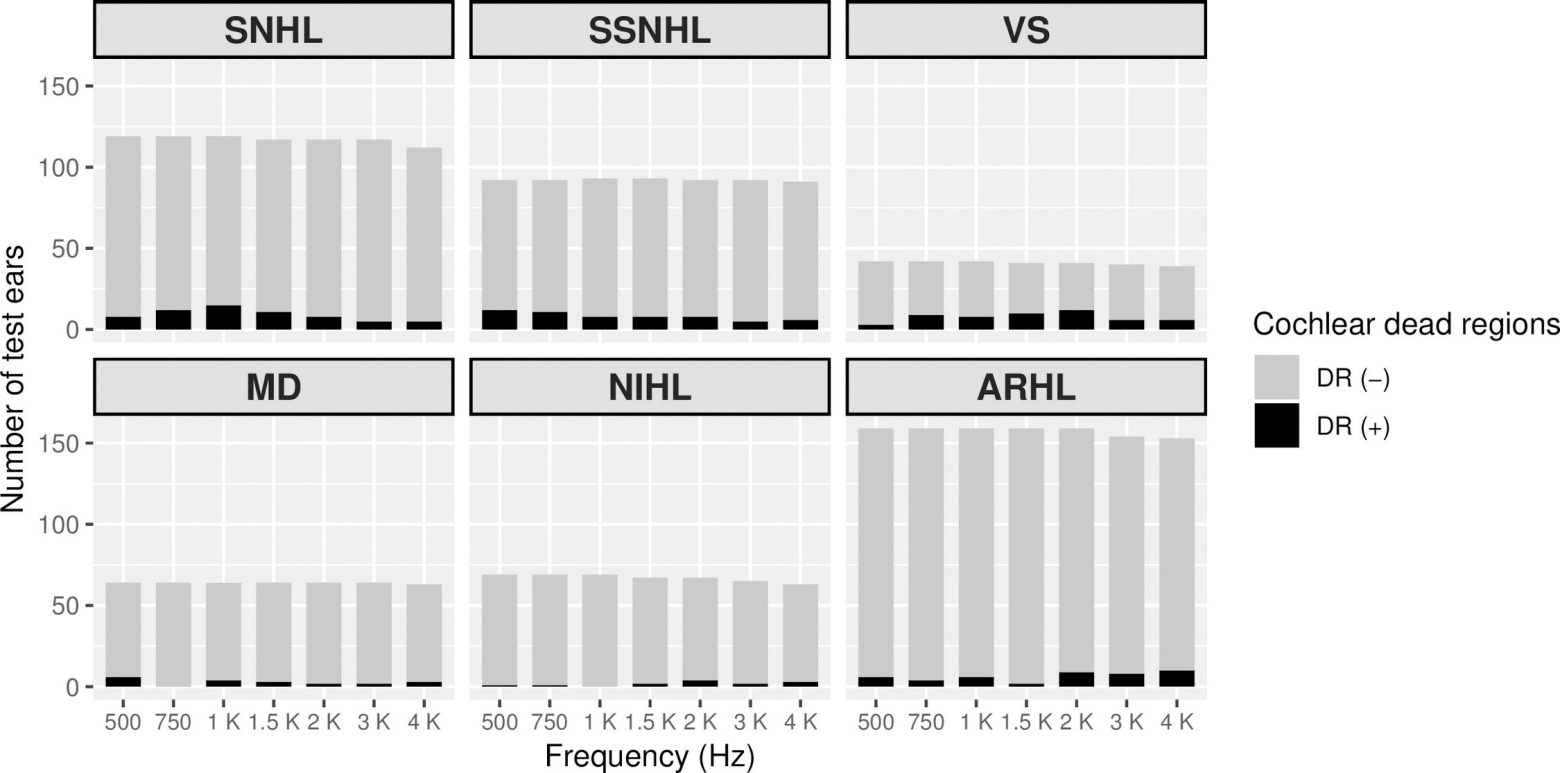
The presence of cochlear dead regions according to the disease type and frequency. The grey bar denotes the number of samples tested and the black bar denotes the number of diagnosed cochlear dead regions. SNHL, sensorineural hearing loss; SSNHL, sudden sensorineural hearing loss; VS, vestibular schwannoma; MD, Meniere’s disease; NIHL, noise-induced hearing loss; ARHL, age-related hearing loss.

The distribution of DRs according to the hearing thresholds of each frequency is illustrated in Fig 1. In Fig 2, the presence of DRs according to disease type and frequency are described. The prevalence of DRs was significantly higher in the VS group, compared to the SNHL with unknown etiology group (p < 0.001, Chi-square test). The other groups were not significantly different from one another.

**Table 1:** Clinical characteristics of the study population.

*Number of patients (380 patients)*	
Age (mean ± SD, range, yr)	56.4 ± 13.8 (20–98)
Sex (Male/Female)	216 (56.842%) / 164 (43.42%)
Test ears	
Unilateral	205 (53.95%)
Bilateral	175 (45.05%)
*Number of test ears (555 ears)*	
Side	
Right	285 (51.35%)
Left	270 (48.65%)
PTA (dB)	44.8 ± 16.0
WRS (%)	82.1 ± 23.9
Types of diseases	
SNHL with unknown etiology	114 (20.54%)
SSNHL	99 (17.84%)
VS	39 (7.03%)
MD	65 (11.71%)
NIHL	70 (12.6%)
ARHL	168 (28.47%)
Number of test samples	
500 Hz	545 (98.20%)
750 Hz	545 (98.20%)
1000 Hz	546 (98.38%)
1500 Hz	541 (97.48%)
2000 Hz	540 (97.30%)
3000 Hz	532 (95.86%)
4000 Hz	521 (93.87%)

Mean pure-tone average (PTA) was calculated for four frequencies (0.5 kHz, 1 kHz, 2 kHz, and 4 kHz). WRS, word recognition score; SNHL, sensorineural hearing loss; SSNHL, sudden sensorineural hearing loss; VS, vestibular schwannoma; MD, Meniere’s disease; NIHL, noise-induced hearing loss; ARHL, age-related hearing loss.

**Fig 3:**
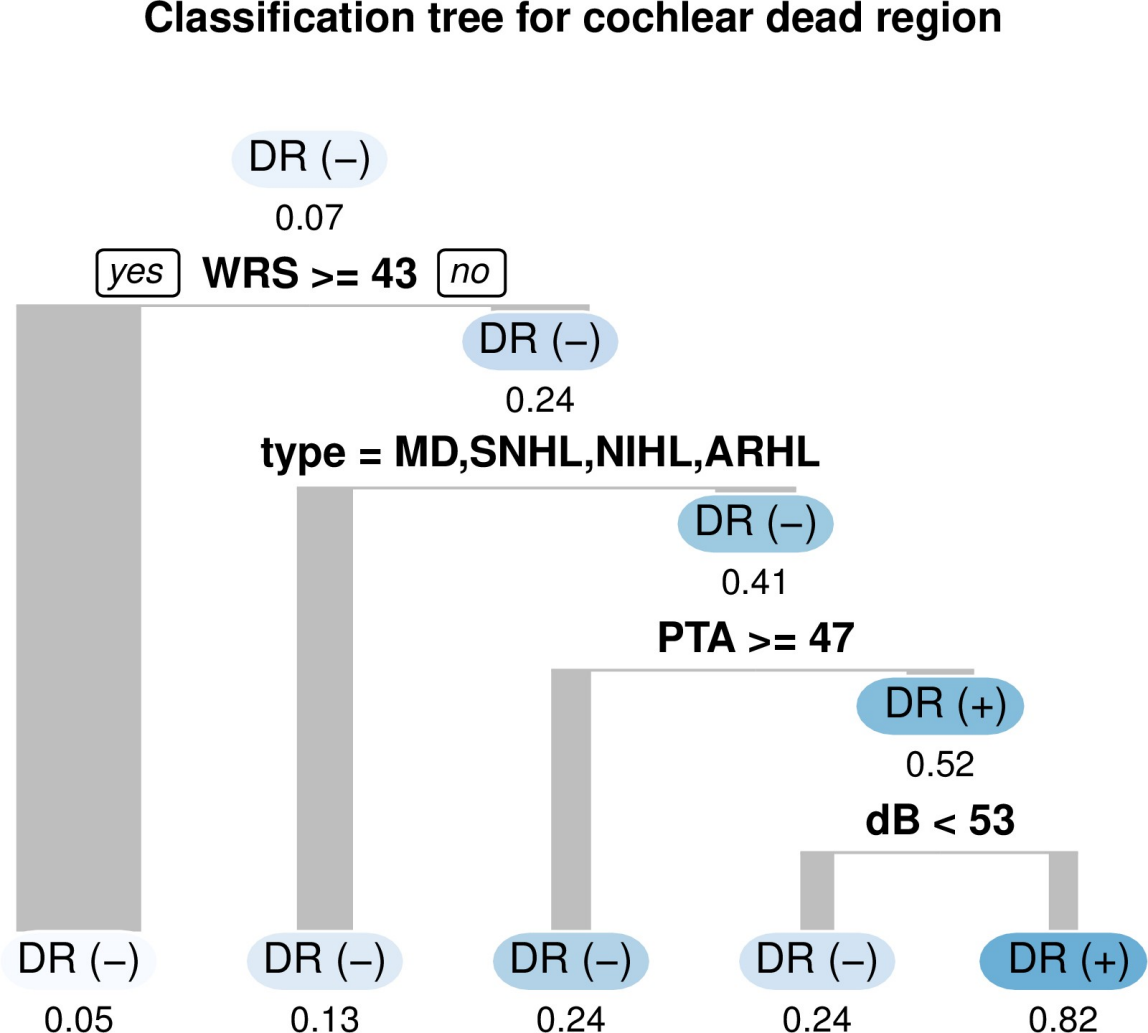
Classification tree model. The word recognition score, etiology types, pure-tone averages, and hearing thresholds at each frequency were used to predict cochlear dead regions. If the condition noted in the midline was satisfied, the patient was moved to the left branch. Branch widths are proportional to the number of observations, and the proportion under the circle represents the ratio of patients with dead regions in each subgroup. The bluer the subgroup node, the higher the ratio of dead regions. DR, cochlear dead region; WRS, word recognition score; SNHL, sensorineural hearing loss; MD, Meniere’s disease; NIHL, noise-induced hearing loss; ARHL, age-related hearing loss; PTA, pure-tone average of four frequencies (0.5 kHz, 1 kHz, 2 kHz, and 4 kHz).

In the CT model, several factors such as WRS (break point: 42%), disease type, pure-tone thresholds at each frequency (break point: 75 dB), and frequency information were used to split the data and detect DRs (Fig 3). Using the WRS break point of 42%, the ratio of DR increases from 0.07 to 0.24. Diagnosis of SSNHL and VS increases the ratio of DR to 0.41. If the overall pure-tone average of four frequencies (0.5 kHz, 1 kHz, 2 kHz, and 4 kHz) (PTA) is higher than 47 dB (poor overall hearing threshold), the ratio of DR is decreased to 0.24; whereas if the hearing threshold of evaluating frequency is higher than 53 dB, the ratio of DR is increased to 0.82. Sex, age, and side were not used in the CT model, which indicates that none of these variables were significant factors for detecting DRs.

The results of multivariate logistic regression analyses for DR detection are shown in Table 2. VS was significantly associated with the presence of DR (odds ratio = 2.40, 95% confidence interval (CI) 1.36–4.23, *p* = 0.002), and MD showed a significantly lower odds ratio than the SNHL group (odds ratio = 0.36, 95% CI 0.18–0.73, *p* = 0.004). The pure-tone thresholds of the evaluating frequencies showed a positive association with DR presence (odds ratio = 1.11, 95% CI 1.09–1.13, *p* < 0.001), whereas the odds ratio for DR presence in the pure tone average was lower than the odds ratio in the control (odds ratio = 0.94, 95% CI 0.92–0.96, *p* < 0.001). The frequencies of 3000 Hz and 4000 Hz showed lower odds ratios than the reference frequency, 1000 Hz (odds ratio = 0.22, 95% CI 0.11–0.46, *p* < 0.001 and odds ratio = 0.31, 95% CI 0.15–0.62, *p* < 0.001, respectively). In contrast to the CT model, the male showed a lower odds ratio than the control (female) for DR prediction (0.42, 95% CI 0.29–0.61, *p* < 0.001).

**Table 2:** Results of multivariate logistic regression analyses for detecting cochlear dead regions.

	Odds ratio	95% confidence interval	P value
Age	0.99	0.98–1.01	0.36
Sex (reference: Female) Male	0.42	0.29–0.61	< 0.001
PTA (dB)	0.94	0.92–0.96	< 0.001
WRS (reference: ≥ 40) < 40	3.77		< 0.001
Pure tone threshold of each frequency (dB)	1.11	1.09–1.13	< 0.001
Types of diseases			
(reference: SNHL)			
SSNHL	1.45	0.88–2.41	0.15
VS	2.40	1.36–4.23	0.002
MD	0.36	0.18–0.73	0.004
NIHL	0.46	0.18–1.15	0.10
ARHL	0.96	0.53–1.74	0.88
Frequency			
(reference: 1000 Hz)			
500 Hz	1.36	0.74–2.53	0.32
750 Hz	1.12	0.60–2.07	0.73
1500 Hz	0.66	0.34–1.26	0.21
2000 Hz	0.82	0.44–1.53	0.53
3000 Hz	0.22	0.11–0.46	< 0.001
4000 Hz1	0.31	0.15–0.62	< 0.001
Intercept	0.01	0.00–0.02	< 0.001

Mean pure-tone average (PTA) was calculated for four frequencies (0.5 kHz, 1 kHz, 2 kHz, and 4 kHz). WRS, word recognition score; SNHL, sensorineural hearing loss; SSNHL, sudden sensorineural hearing loss; VS, vestibular schwannoma; MD, Meniere’s disease; NIHL, noise-induced hearing loss; ARHL, age-related hearing loss.

**Fig 4:**
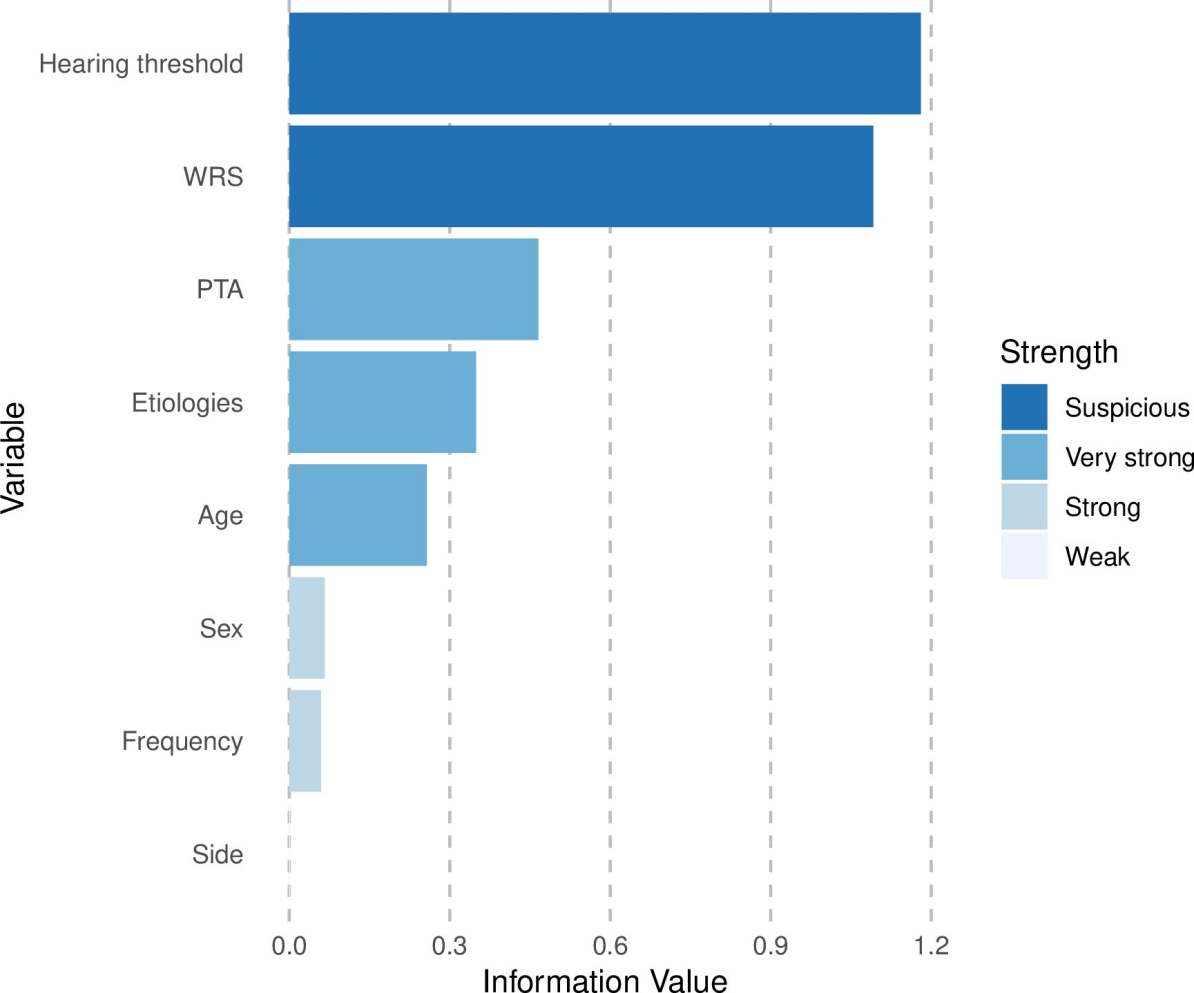
Plot of the variable importance with total decrease in node impurity as obtained by random forest. The variables are presented according to higher informative value (brown) or lower informative value (peach). WRS, word recognition score; PTA, pure-tone average of four frequencies (0.5 kHz, 1 kHz, 2 kHz, and 4 kHz).

In the RF model, variable importance was calculated and is presented in Fig 4. The hearing thresholds of the evaluating frequencies and WRS are the most informative variables for splitting the data with higher ratios of DR. On the other hand, side, frequency, and sex were less informative.

**Fig 5:**
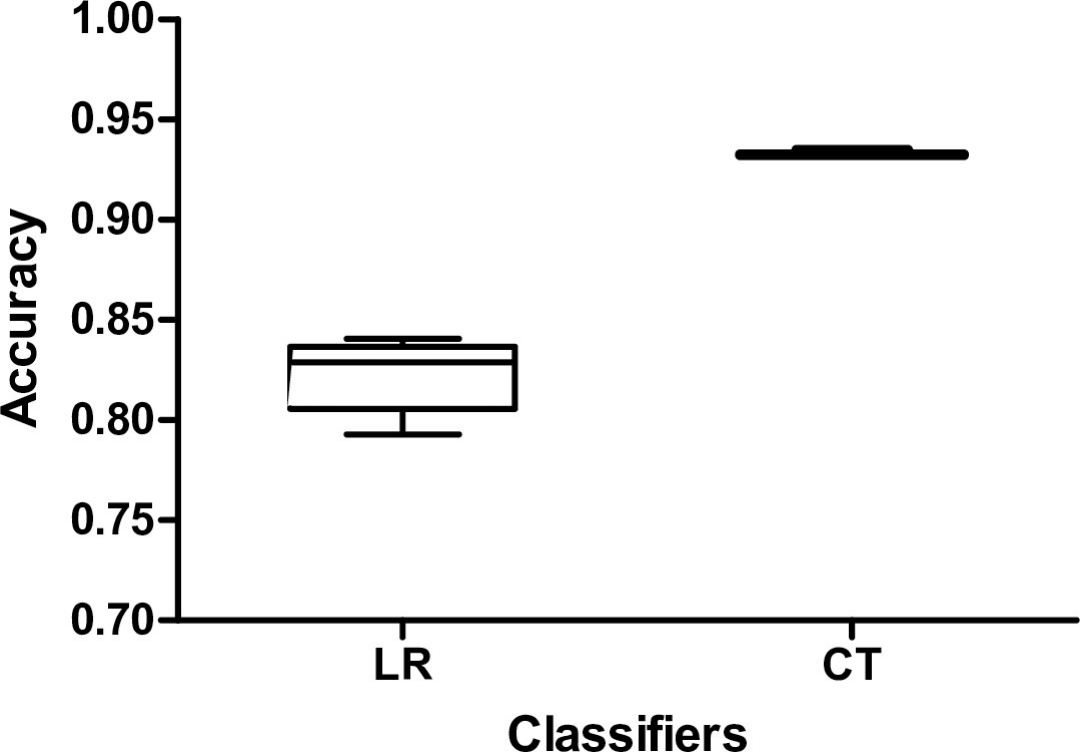
Performance of various models on prediction of cochlear dead regions. LR, logistic regression; CT, classification tree.

When model accuracy was calculated with the test data in the CT model, the PPV was 62.08%, and the NPV was 94.49%. The LR model had a PPV of 21.60% and an NPV of 96.50%. The RF model had a PPV of 59.09% and an NPV of 95.17%. The sensitivities of LR, CT, and RF were 59.74%, 23.38%, and 33.77%, respectively. The accuracy results of 10-fold cross-validation of LR and CT were 0.82 (± 0.02) and 0.93 (± 0.01), respectively (Fig 5). The performance of the three models is summarized in Table 3.

**Table 3:** Predictive performance of the machine-learning models.

Model	Positive predictive value	Negative predictive value	Sensitivity	Specificity
Classification tree	62.07%	94.49%	23.38%	98.92%
Logistic regression	21.60%	96.50%	59.74%	83.66%
Random forest	59.09%	95.17%	33.77%	98.24%

## Discussion

Detection of DRs is the first step in understanding the clinical importance of DR. One of the biggest hurdles for evaluating the role of DRs is that the TEN (HL) test is very time consuming. Although previous studies have revealed reliable indicators of DRs based on detection by TEN (HL) tests [2, 11, 12], the prevalence and possible indicators of DRs differ according to the study population [2, 12, 25, 26]. Therefore, it is still unclear which patients beyond those with severe-to-profound hearing loss should undergo the TEN (HL) tests. There have been no previous studies that specifically address DR prediction as a function of frequency-specific information.

The ML-based approach provided well-validated and ready-to-use prediction models for clinical practitioners. Using an ML-based approach, we developed and validated DR prediction models as a function of frequency. The prediction models were built with several ML techniques: CT, LR, and RF models. This is also the first study to evaluate DR as a function of frequency. Using these prediction models, we achieved PPVs of 21.60–62.07%. Although the PPV in the LR model was lower than that of the CT or RF model, the sensitivity of the LR model was 59.74% as a function of frequency. This result indicates that the LR model could potentially serve as a screening model to detect the most DRs with the highest sensitivity among the three different models. Although the accuracy of the LR model is lower than those of the other models, we achieved a higher sensitivity by setting the predictive probability of DRs to 0.1. Considering that the present study evaluated DRs according to frequency with an appropriate screening model, the sensitivity of the LR model is significant for clinical applications. Previous studies [1, 26] only assessed the prevalence of DRs by ear, not by frequency, which indicates that it is difficult to predict or detect DRs according to frequency in clinical settings.

In addition, we observed that there are large differences between the PPV of the LR model and the PPVs of the CT and RF models. Considering that the CT and RF models are more suitable for non-linear data structures, the large differences obtained in this study imply that the presence of DRs in the study population follows a non-linear distribution or that there are unknown indicators of DRs, such as duration of hearing loss, genetic polymorphisms [27], and a history of ototoxic drug use [28]. Therefore, addressing these potential factors may be necessary to improve the predictive power of the models.

Because this study included patients with diverse etiologies and wide ranges of hearing loss, the indicators identified here may be beneficial for determining which patients have suspected DRs. WRS, etiology types, and hearing thresholds at each frequency are informative factors in the three different models. WRS, which has been addressed in a previous study [29], can be a useful indicator for predicting DRs. In the CT model, the 43% value used for classifying the break point in WRS was suggested, but the cut-off value may vary according to study population. VS patients showed higher prevalence, and therefore VS can be used as a possible predictive factor for determining the presence of DRs. A previous study had reported that VS-secreted extracellular vesicles are a major contributing factor in selective cochlear nerve damage and can lead to SNHL [30]. Cochlear nerve damage may be associated with the presence of DR. In addition, previous studies have suggested that hearing loss in patients with VS may be the result of secondary cochlea damage [31, 32] or ischemia. Degeneration of the inner and outer hair cells, striae vascularis, and the spiral ligament have been documented in cases of vestibular schwannoma [33]. Therefore, the presence of DRs reflects poor inner hair cell function secondary to VS. In MD, the presence of DR is lower and negatively associated in both the CT and LR models. This result is consistent with that of a previous study [26]. Considering that endolymphatic hydrops plays a key role in hearing loss and that inner hair cell damage occurs in late stage MD, DRs are not commonly detected in MD, and thus we can use MD as a negative indicator of DRs.

In contrast to previous studies [2, 26], the feature “high frequencies” was negatively associated with DRs in the present LR model. This result may depend on the study population. Our study enrolled ARHL and NIHL patients, and these populations show a low prevalence of DRs, despite poorer hearing thresholds at high frequencies.

This study has some limitations. First, because the possible risk factors for DR are not fully understood, we could not assess all possible causes. This may affect the predictive powers of the ML models. Second, we used the TEN (HL) test to detect DR. The TEN (HL) test is a well-described and reliable subjective test; however, if we adopt objective tests, such as an electrophysiologic approach using the acoustic change complex, the results could differ [34]. In addition, the psychophysical tuning curve is accepted as a more reliable test for detecting DRs. However, in this study, we aimed to make a predictive model for DRs based on the TEN (HL) test for usage in clinical practice. Third, we only included SNHL patients. Therefore, neither the prediction model nor the indicators can be extended to conductive hearing loss patients.

Nevertheless, our study also possesses sufficient strength to support several results. This study is the first to address and develop DR predictions using frequency models and several ML techniques. WRS, etiology types, and hearing thresholds at each frequency were revealed to be informative factors. This result can be adopted to screen eligible patients with the TEN (HL) test. Further investigation for data pre-processing to apply diverse non-linear models will be needed to improve the model performance for predicting DR.

## Supporting information

S1 File Detailed numeric data in this study. (CSV)
